# CRISPR-Cas9 screening develops an epigenetic and transcriptional gene signature for risk stratification and target prediction in neuroblastoma

**DOI:** 10.3389/fcell.2024.1433008

**Published:** 2024-08-08

**Authors:** Liaoran Zhang, Jialin Mo, Hao Shi, Jing Xiong, Yeerfan Aierken, Feng Chen, Yujie Tang, Kewen Zhao, Zhibao Lv, Kezhe Tan

**Affiliations:** ^1^ Department of General Surgery, Shanghai Children’s Hospital, School of Medicine, Shanghai Jiao Tong University, Shanghai, China; ^2^ Shanghai Key Laboratory of Reproductive Medicine, Department of Histoembryology, Genetics and Developmental Biology, School of Medicine, Shanghai Jiao Tong University, Shanghai, China; ^3^ State Key Laboratory of Oncogenes and Related Genes, Key Laboratory of Cell Differentiation and Apoptosis of National Ministry of Education, Department of Pathophysiology, School of Medicine, Shanghai Jiao Tong University, Shanghai, China

**Keywords:** neuroblastoma, epigenetic and transcriptional genes, prognostic model, MYCN, drug targets

## Abstract

**Objectives:** Neuroblastoma (NB), a pediatric malignancy of the peripheral nervous system, is characterized by epigenetic and transcriptional (EP-TF) anomalies. This study aimed to develop an EP-TF clinical prognostic model for NB using CRISPR-Cas9 knockout screening.

**Results:** An integrative analysis was conducted using CRISPR-Cas9 screening *in vitro* and *in vivo* with public NB datasets to identify 35 EP-TF genes that exhibited the highest expression in NB and were highly dependent on cancer viability. After univariate analysis, 27 of these 35 genes were included in the least absolute shrinkage and selection operator screen. We established and biologically validated a prognostic EP-TF model encompassing *RUVBL1, LARP7, GTF3C4, THAP10, SUPT16H, TIGD1, SUV39H2, TAF1A, SMAD9,* and *FEM1B* across diverse NB cohorts. MYCN serves a potential upstream regulator of EP-TF genes. The high-risk subtype exhibited traits associated with the malignant cell cycle, MYCN-linked signaling and chromatin remodeling, all of which are correlated with poor prognosis and immunosuppression. MEK inhibitors have emerged as promising therapeutic agents for targeting most EP-TF risk genes in NB.

**Conclusion:** Our novel prognostic model shows significant potential for predicting and evaluating the overall survival of NB patients, offering insights into therapeutic targets.

## Introduction

Neuroblastoma (NB), the most prevalent extracranial solid tumor in children, constitutes more than 6% of childhood cancers (Siegel et al., 2024). Based on the classification criteria of the Children’s Oncology Group, patients are stratified into very low-, low-, intermediate- and high-risk groups, with high-risk status indicated by amplification of the *MYCN* proto-oncogene (Irwin et al., 2021). Despite advancements in treatment modalities, the 5-year event-free survival rate for patients with high-risk NB (HR-NB) is less 60%, representing approximately half of all NB cases (Munoz et al., 2023). Consequently, the critical aim of NB research is to enhance the survival and quality of life of patients with HR-NB.

HR-NB, a neuron-derived pediatric cancer, is distinguished by epigenetic and transcriptional aberrations (Gartlgruber et al., 2020). Altered epigenetic and transcriptional (EP-TF) mechanisms include DNA methylation, histone modification, noncoding RNA regulation, super-enhancer modification, bromodomain regulation and chromatin remodeling, particularly in *MYCN*-amplified NB (Jimenez et al., 2023). Moreover, *MYCN* is a critical transcription factor that promotes cell cycle progression by targeting *CDK4, CHK1, ID2, MCM, MYBL2, SKP2,* etc., and influences a broad transcriptional network that is still under investigation (Matthay et al., 2016). This highlights the necessity of identifying additional epigenetic and transcription biomarkers to refine clinical assessments. HR-NB cancer also exhibits low T-cell infiltration and impaired natural killer (NK) cell activity, leading to an immunosuppressive tumor microenvironment (Yang et al., 2023).

To address these challenges, we conducted an integrative analysis using CRISPR-Cas9 knockout screening and other NB transcriptomic methods to develop an EP-TF gene signature, providing a new diagnostic and prognostic tool. We assessed the biological and clinical relevance of the EP-TF signature in the training set (TS), internal validation set (IVS) and external validation sets (EVSs), complemented by preliminary experimental validation in NBs from our center. Furthermore, we examined immune cell infiltration in HR-NB and identified potential drug targets in the Genomics of Drug Sensitivity in Cancer (GDSC) database. Our findings reveal a novel set of EP-TF biomarkers for managing NB, and suggest that MEK inhibitors (MEKis) may effectively target key EP-TF associated genes.

## Methods and materials

### Ethics declaration

The study received approval from the Institutional Review Board at Shanghai Children’s Hospital (SCH) of Shanghai Jiao Tong University and was conducted according to the guidelines of the Declaration of Helsinki. Written informed consent was obtained from the legal guardians or next of kin of the participants. All necessary steps were taken to maintain confidentiality and protect the privacy of the patients. Furthermore, the Medical Experimental Animal Administrative Committee in Shanghai sanctioned all protocols related to animals.

### Patients and specimens

Sixty primary NB specimens, classified according to the Children’s Oncology Group’s risk criteria, were collected at SCH from January 2015 to December 2019. These samples were immediately frozen in liquid nitrogen and subsequently stored at −80°C.

### Cell culture

All experiments were performed with mycoplasma-free cells. HEK293T (RRID:CVCL_0063) cells for virus packaging were obtained from Zhao’s lab and cultured in DMEM (BasalMedia, #L110KJ) supplemented with 10% fetal bovine serum (FBS; Sigma, #F2442) and 1 × penicillin/streptomycin (P/S) solution (BasalMedia, #S110JV). The *MYCN*-amplified NB cell lines, including BE (2)-C (RRID:CVCL_0529), SK-N-BE2 (RRID:CVCL_0528) and IMR-32 (RRID:CVCL_0346) were acquired from the Cell Bank of the Chinese Academy of Sciences. BE (2)-C and SK-N-BE2 cells were grown in DMEM/F12 (Gibco, #11330-032) supplemented with 10% FBS and 1 × P/S. IMR-32 cells were cultured in MEM (BasalMedia, #L510KJ) supplemented with 10% FBS and 1×P/S. The authenticity of the NB cell lines was confirmed through short tandem repeat sequencing (BIOWING Biotech Co., Ltd., Shanghai) (Supplementary Table S1).

### Animal experiments

Female BALB/c nude mice (4–6 weeks old) were acquired from Experimental Animal Center of the Chinese Academy of Sciences in Shanghai. For the subcutaneous xenograft model, either 5 × 10^6^ *MYCN*-amplified NB cells or 4.25 × 10^6^ Cas9-sgRNA-library transfected cells were implanted subcutaneously into the dorsal flanks of the mice. Tumor volumes were calculated using the formula 1/2 (long axis * short axis^2). Mice with tumors exceeding 1,500 mm^3^ were humanely euthanized. A few NOD/SCID/gamma mice were purchased from GemPharmatech Co., Ltd. (Jiangsu, China), and the same subcutaneous xenograft procedures were used.

### CRISPR-Cas9 knockout screening

CRISPR-Cas9 screening was conducted as described previously (Mo et al., 2021; Tan et al., 2023). Briefly, BE (2)-C cells were transduced with lentiCas9-Blast (Addgene, #52962) at a multiplicity of infection (MOI) of less than 0.7 to establish Cas9-expressing cell lines. These cells were subsequently transduced with an sgRNA library targeting EP-TF regulatory genes at an MOI of less than 0.3. The library contained 16,408 sgRNAs covering 2,771 EP-TF regulatory genes (Supplementary Table S2). After library transduction for 2 days, the cells were selected with 1.25 μg/mL puromycin for 4 days. It was estimated that 10% of the xenografted cells would survive *in vivo*, and 500 × coverage of the sgRNA library was needed; therefore, 4.25 × 10^6^ cells were implanted subcutaneously into each side of the dorsal flanks of 10 mice, while an additional 8.5 × 10^6^ sgRNA library-transduced cells were maintained *in vitro*. After 28 days, in vivo-generated tumor cells were collected and homogenized, and in vitro-generated cells were harvested for genomic DNA isolation. At least 8.5 × 10^6^ cells per group were collected for genomic DNA isolation to maintain at least 500 × coverage of the sgRNA library. A 2 × KAPA HiFi kit (Roche, #KK2602) was used to amplify the sgRNA sequences, and the PCR products were separated with FastDigest loading buffer (Thermo Fisher, B72) on a 2% agarose gel by DNA electrophoresis. The objective DNA bands were harvested and repurified with a FastPure Gel DNA kit (Vazyme, DC301-01). Eventually, the quantified PCR products were sequenced by Novogene Technology (Beijing, China), and the sgRNA read counts were analyzed using Python 2.7 and the MAGECK algorithm (Li et al., 2014) to assess the viability screen *in vitro* and *in vivo* (Supplementary Table S3).

### Virus packaging and cell transfection

Lentiviral sgRNA plasmids were constructed by cloning target oligonucleotides into lentiGuide-Puro (Addgene, #52963) vectors. Plasmid DNA was extracted from Vazyme (DC112-01) using a kit. Lentiviruses were produced by co-transfecting HEK293T cells with plasmids containing packaging vectors (psPAX and pMD2.G) and PEI MAX solution (Polysciences, #24765). The viral supernatant was then collected, filtered through a 0.45 μm strainer, concentrated with PEG6000 (Sigma, #81253), and prepared in PBS for further transfections. Infected cells were selected for 72 h using either blasticidin (for the selection of Cas9 transfected cells) (20 μg/mL, YEASEN, 60218ES60) or puromycin (for the selection of Cas9-sgCDK7- or Cas9-sgRNA-library-transfected cells) (1.25 μg/mL, YEASEN, 60210ES25).

### Immunoblotting

Cells were lysed in RIPA buffer (Thermo Fisher Scientific, #89900), and protein concentration was determined using a Pierce BCA kit (Thermo Fisher Scientific, #23225). Denatured proteins (10–20 μg/lane) were separated via sodium dodecyl sulfate polyacrylamide gel electrophoresis and subsequently transferred to polyvinylidene difluoride membranes. The membranes were then blocked with 5% nonfat milk (BD Biosciences, #232100) in Tris-buffered saline containing Tween 20 (TBST) and incubated overnight with the following primary antibodies: mouse anti-human Flag (1:500; Sigma, #F1804), mouse anti-human CDK7 (1:1000; Cell Signaling Technology, #2916) or rabbit-anti-human β-tubulin (1:5000; Abcam, #ab6046). HRP-linked secondary antibodies were used for detection (goat anti-rabbit/mouse IgG (0.2 μg/mL; Pierce, #31460/#31430)). Band visualization was performed using a luminescent image analyzer (Fujifilm, LAS-4000) following incubation with enhanced chemiluminescence reagents (Millipore, WBKLS0500).

### Quantitative reverse transcription PCR (Q-RT-PCR)

Approximately 2.5 × 10^5^ cells were lysed with TRIzol (Thermo Fisher Scientific, #TR118) to extract total RNA. This RNA was then reverse transcribed to cDNA using a High-Capacity RNA-to-cDNA kit (Thermo Fisher Scientific, #4387406). Quantitative PCR was performed using a QuantStudio™ 5 Real-Time PCR System (Thermo Fisher Scientific, #A34322) with SYBR Green Master buffer (ROX) (Thermo Fisher Scientific, #A25742). GAPDH served as the reference gene, and mRNA levels were quantified using the 2^delta Ct method. The primer sequences are available in Supplementary Table S4.

### Cell viability assay

Cells were seeded in triplicate in 96-well plates at a density of 1,000 cells/100 μL of medium. Cell viability was assessed using the CellTiter-Glo^®^ luminescent cell viability assay (Promega, #G7573) on days 0 and 3 following the provided protocol.

### Public data acquisition

Over 50 publicly available NB datasets were reviewed. Our criteria prioritized RNA sequencing (RNA-seq) over microarray for transcriptome analysis due to the heightened sensitivity of RNA-seq to low abundant transcripts and better suitability for immune infiltration studies (Zhao et al., 2014). The inclusion criteria for TS were that the NB RNA-Seq samples should be of human origin with at least 200 samples and have detailed survival data. The exclusion criteria ruled out NB patients with concurrent diseases. Given these considerations, we selected the NB RNA-seq dataset GSE62564 (n = 498), which has the largest sample size and the most comprehensive clinical and survival information, for our analysis. The dataset was used for gene set enrichment analysis (GSEA), gene signature development, etc. It was randomly divided into a TS (n = 349) and an IVS (n = 149) at a 7:3 ratio.

For the external validation datasets, the criteria required a transcriptome depth comparable to that of the TS dataset and excluded samples from patients with other diseases. Therefore, we used the NB RNA-seq dataset EGAS00001001308 (referred to as EGAS) and microarray datasets (GSE16476 and GSE85047) from the R2 database (https://hgserver1.amc.nl/cgi-bin/r2/main.cgi). Additionally, microarray data from the GPL570 platform, including normal adrenal gland (GSE3526, GSE7307 and GSE8514) and HR-NB tissue (GSE12460, 13136 and 16476) data, were retrieved. Cancer cell line encyclopedia data (version 22Q1) were downloaded from https://depmap.org/portal/.

In addition, we acquired NB patient derived xenografts (PDXs) datasets including GSE90121 and GSE120920 to facilitate detailed analyses. To elucidate the potential mechanisms, we utilized chromatin data (GSE94822) and MYCN knockdown datasets (GSE80397, GSE121529 and GSE132760). For prediction of therapeutic targets, we obtained datasets concerning MEKis (GSE115406 and GSE130401).

### Identification of differentially expressed genes, Venn diagram and functional enrichment

Differentially expressed genes between two groups were identified using a *t*-test, with a cutoff of *p* < 0.05 and an absolute log2 (fold change, FC) > 1. Venn diagram analysis was conducted using an online tool (http://bioinformatics.psb.ugent.be/webtools/Venn/) to identify common EP-TF genes. The “clusterProfiler” package in R was used for GO and KEGG pathway enrichment analysis of the EP-TF genes. GO terms were categorized into biological process (BP), cellular component (CC) and molecular function (MF) categories. The top 5 enriched items are illustrated in the figures.

### Protein-protein interaction (PPI) network and gene dependency analysis

The STRING database (http://string-db.org/) was used to construct a PPI network for the EP-TF genes with a minimum interaction score threshold of 0.15. The network was analyzed and visualized using Cytoscape (version 3.8.2) with the CytoHubba plugin, which identifies hub genes. Further gene dependency analyses were conducted using data from the DepMap portal (https://depmap.org/portal/). Additionally, gene-gene correlations were assessed using the Pearson correlation coefficient.

### Establishment of a prognostic gene signature

A total of 498 patients from the GSE62564 dataset were included in the analysis. Initially, 35 EP-TF genes were subjected to univariate Cox regression analysis to identify prognostic genes (*p* < 0.05) across the entire dataset. The cohort was then split into a TS and an IVS using a 7:3 distribution. A new prognostic model encompassing multiple genes was developed by performing LASSO regression analysis on the prognostic EP-TF genes using the “glmnet” package in R. The LASSO risk score (LRS) was used to estimate prognosis as follows: risk score = $\sum_{i=1}^{n}\left(\text{Coefi }x\text{ Genei}\right)$ .

### Chromatin immunoprecipitation sequencing (ChIP-seq) analysis

Anti-MYCN ChIP-seq data on BE (2)-C and Kelly MYCN-amplified NB cells (GSE94822) were downloaded, mapped against the human genome build hg19 and then visualized using Integrative Genomics Viewer (version 2.16.0).

### Clinical assessment of the gene signature in NB

Patients with NB were categorized into high-risk and low-risk groups according to the median LRS derived from TS, IVS and EVSs. The prognostic value of the gene signature within TS, IVS and EVSs were analyzed and visualized based on Kaplan-Meier curves (the package “survival,” “survminer,” and “ggplot2” in R) and time-dependent receiver operating characteristic (ROC) curves (package “timeROC” in R). The hazard ratio (HR) was used to determine risk factors, with an HR > 1 indicating increased risk and an HR < 1 indicating protective factors.

To ascertain the independence of the EP-TF signature from other clinical parameters such as age, sex, INSS stage, and *ALK, MYCN* and *TERT* levels, both univariate and multivariate Cox analyses were conducted (package “survival” in R). Significant predictors from multivariate analysis were used to create a nomogram and calibration plot, aiding in the prediction of 3-, 5-, and 7-year overall survival (OS) rates for NB patients (package “rms” in R).

### GSEA

GSEA software (version 4.3.2) was used to explore differences in gene expression profiles between the high-LRS and low-LRS groups, focusing on Hallmark gene sets (50 sets, v7.5), *MYCN*-associated gene sets (4 sets, v7.5) and EP-TF associated GO terms (275 sets, v7.5) across diverse cohorts. Statistical significance was determined by a NOM-p value <0.05 or a false discovery rate <0.25.

### Assessment of immune cell subpopulations

Immune cell subpopulations within samples were quantified using the ImmuCellAI tool (http://bioinfo.life.hust.edu.cn/ImmuCellAI#!/). The tool employed the single-sample GSEA algorithm to assess enrichment scores and detect the presence of various immune subsets in individual samples. Furthermore, both immunoactive and immunosuppressive gene signatures were obtained from the tracking Tumor Immunophenotype Database (http://biocc.hrbmu.edu.cn/TIP/).

### Drug sensitivity analysis

The analysis incorporating risk genes implicated in the prognostic model (*RUVBL1*, *GTF3C4*, etc.), as well as the key oncogenes *MYCN, EZH2* and *SMC3*, was conducted using the GDSC database (https://www.cancerrxgene.org/), with analytical support from GSCALite (http://bioinfo.life.hust.edu.cn/GSCA/#/). The approach for predicting drug targets employed multiple algorithms as outlined in the extended method section (https://www.cancerrxgene.org/gdsc1000/GDSC1000_WebResources//Home_files/Extended%20Methods.html#17).

### Statistical analyses

Data analysis and graphical presentations were generated using R software v4.2.1, GraphPad Prism v9.2.0 and GSEA v4.3.2. Unless noted otherwise in the specific methods mentioned above, statistical significance was determined based on a *p*-value threshold of <0.05.

## Results

### CRISPR-Cas9 knockout screening reveals key tumor-dependent EP-TF genes in NB

**FIGURE 1:**
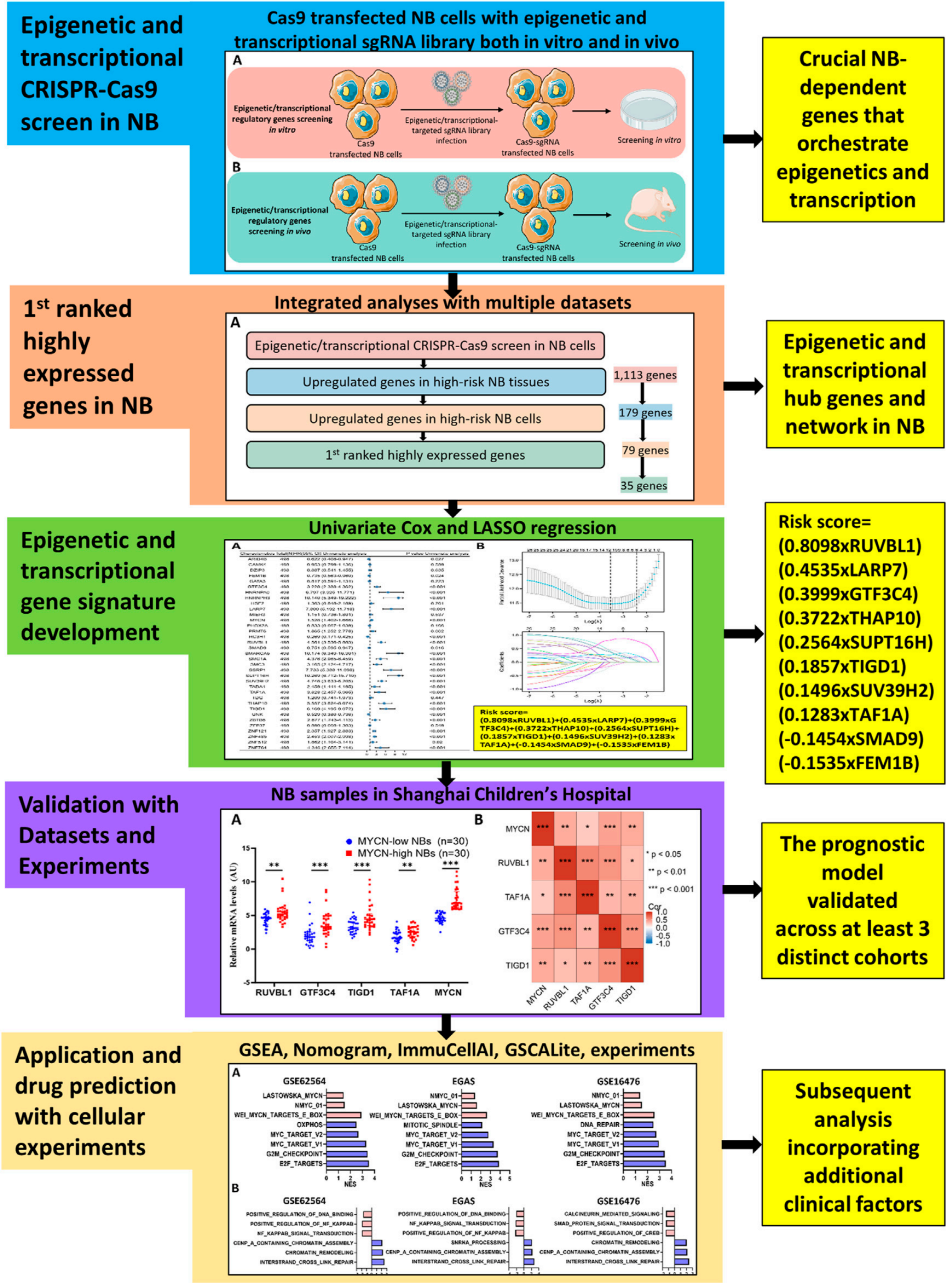
Flowchart of the entire analysis.

**FIGURE 2:**
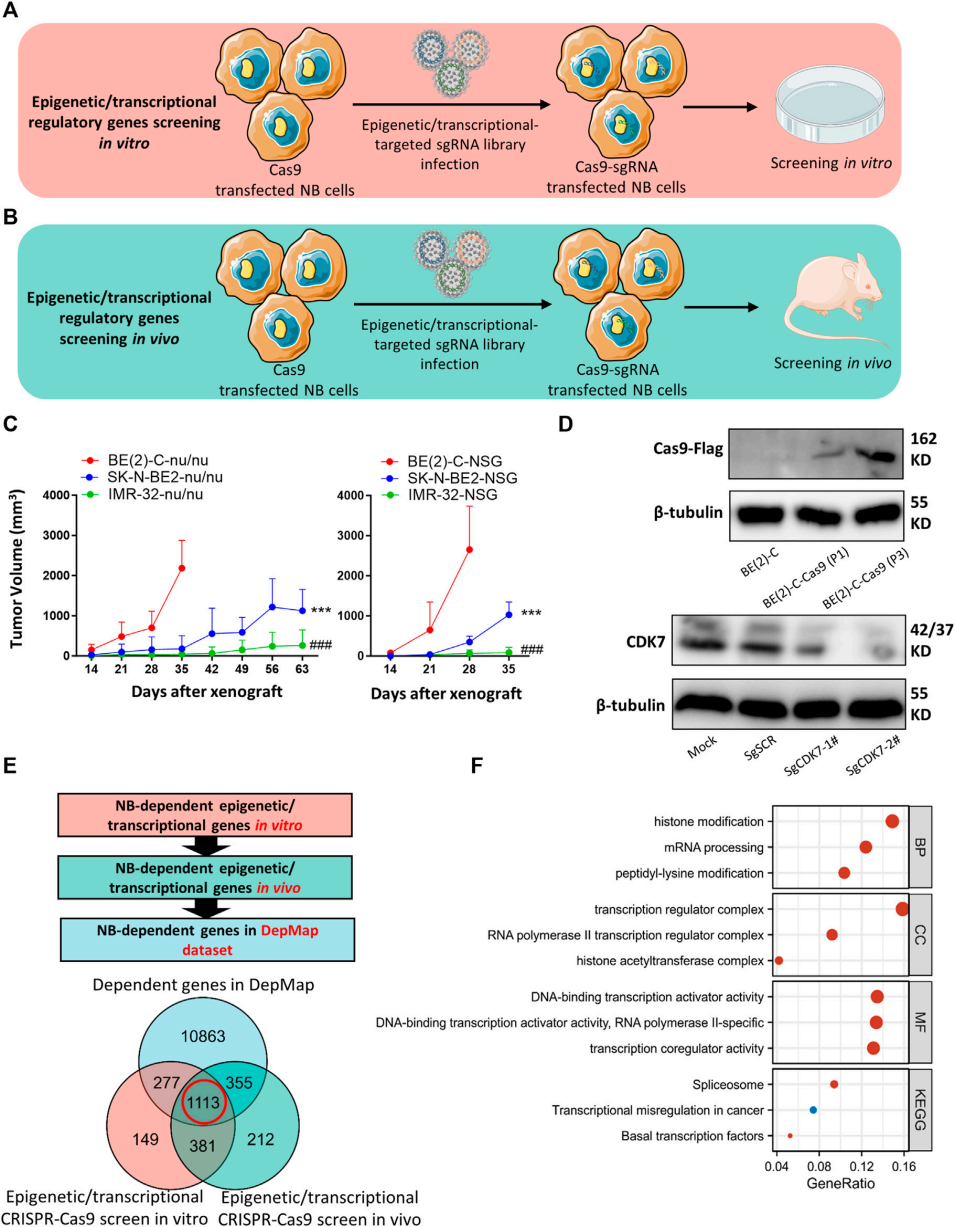
CRISPR-Cas9 knockout screening identifies NB-dependent EP-TF regulatory genes. **(A, B)** Schematic diagrams depicting the methodology used for CRISPR-Cas9 knockout screening targeting EP-TF regulatory genes *in vitro* **(A)** and *in vivo* **(B)**. **(C)** *MYCN*-amplified NB cell growth curve in nude (left panel) and NOD SCID gamma mice (right panel). ^*^ indicates a comparison between BE (2)-C and SK-N-BE2. ^#^ indicates a comparison between BE (2)-C and IMR32. **(D)** Immunoblotting assays verifying Cas9 transfection in BE (2)-C cells (upper panel) and sgCDK7 transfection in BE (2)-C-Cas9 cells (lower panel). **(E)** Integrated analyses of EP-TF regulatory gene screening and the DepMap dataset. **(F)** GO/KEGG analysis of 1,113 genes involved in EP-TF regulation. EP-TF: epigenetic and transcriptional; NB: neuroblastoma; Nu: nude mice; sgRNA: small guide RNA; sgSCR; sg scramble control. *p* < 0.05 is shown as */^#^, *p* < 0.01 as **/^##^ and *p* < 0.001 as ***/^###^.

A detailed map of our analytical process is provided in Figure 1. In an effort to systematically analyze tumor-dependent EP-TF genes in NB, we conducted a comprehensive CRISPR knockout screen targeting EP-TF regulatory factors *in vitro* and *in vivo* (Figures 2A,B). Our focus was on three types of *MYCN*-amplified NB cells (BE (2)-C, SK-N-BE2 and IMR32), and BE (2)-C cells exhibited the highest proliferation rates both *in vitro* (Supplementary Figure S1A) and *in vivo* (Figure 2C). Consequently, we generated stably Cas9 transfected BE (2)-C cells and verified their functional efficacy (Figure 2D). Our CRISPR-Cas9 screening revealed 1,920 (Supplementary Figure S1B) and 2,061 (Supplementary Figure S1C) EP-TF genes crucial for NB *in vitro* and *in vivo*, respectively, with an overlap of 1,494 genes (Figure 2E). By integrating these data with publicly available genome-scale CRISPR knockout data from the DepMap project, we ultimately identified 1,113 EP-TF NB-dependent genes as pivotal for NB dependency (Figure 2E). Subsequent functional enrichment analysis revealed that histone modification, transcription regulator complex, DNA-binding transcription activator activity and spliceosome were the most significant terms in an analysis of the GO-BP, GO-CC, GO-MF and KEGG analyses, respectively (Figure 2F). Overall, our comprehensive CRISPR-Cas9 knockout screen revealed a substantial number of EP-TF genes critical for NB progression.

### High expression profiles highlight 35 hub EP-TF genes in HR-NBs

**FIGURE 3:**
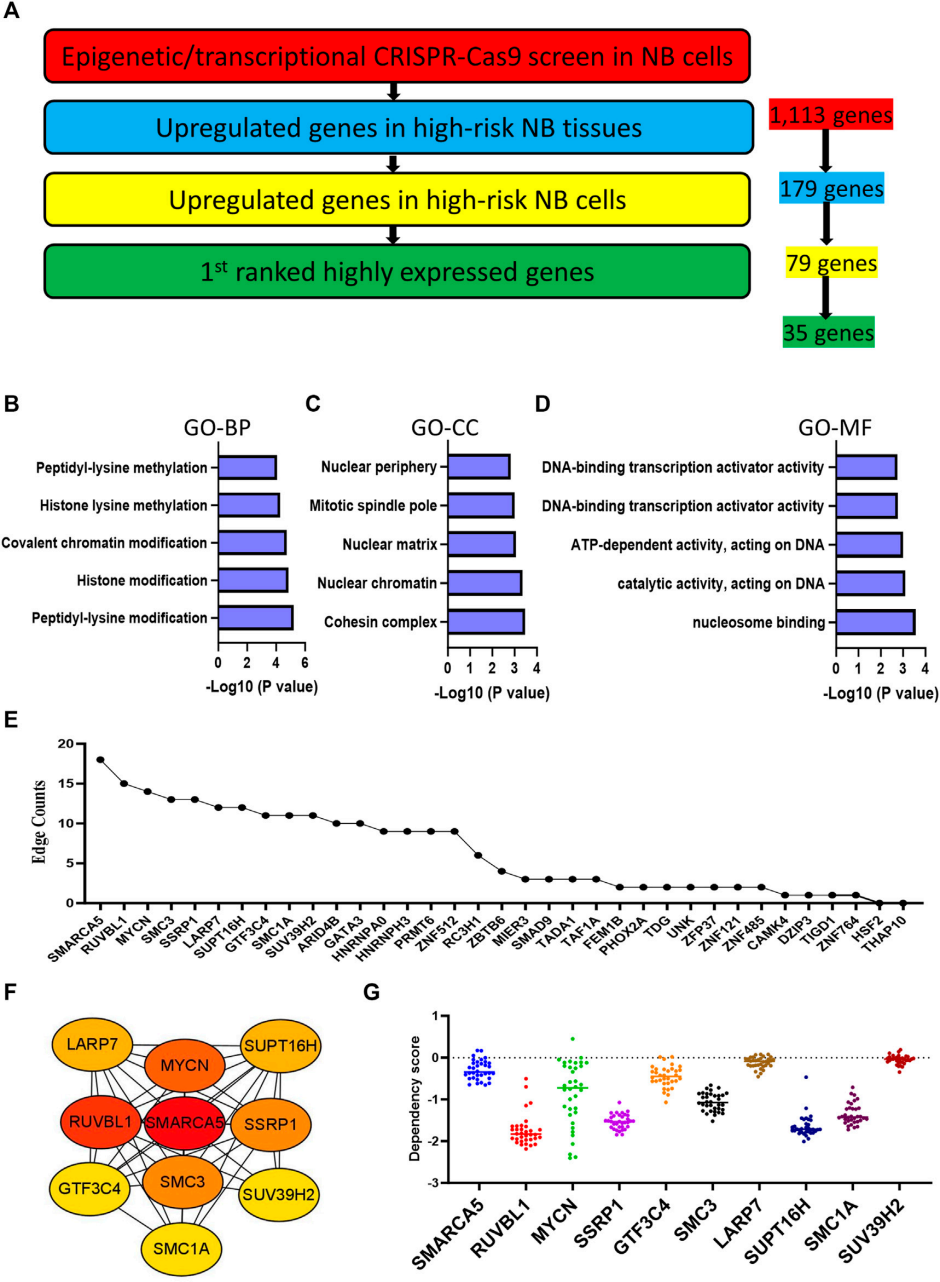
Integration with expression profiling narrows the focus to 35 hub genes. **(A)** Determination of crucial EP-TF regulatory genes in NB. The red section highlights NB-dependent EP-TF genes in our sgRNA library and 1,113 genes are extracted. The blue section shows the significantly upregulated genes in HR-NB tumor tissues compared with normal adrenal gland tissues in all three tested GEO datasets (log2FC > 1, *p* < 0.05 for the NB tissue datasets GSE12460, GSE13136 and GSE16476 versus the normal adrenal gland datasets GSE3526, GSE7307 and GSE8514) and 179 genes are extracted. The yellow section indicates genes whose expression was significantly upregulated in NB cell lines (GSE28019) compared with that normal neural crest cells (GSE14340) (log2FC > 1, *p* < 0.05) and 79 genes are extracted. The green section indicates the first ranked genes in the pan-cancer analysis from the DepMap portal and 35 genes are extracted. **(B–D)** Top five gene sets enriched in functional categories according to GO-BP **(B)**, GO-CC **(C)** and GO-MF **(D)** annotations. **(E)** Ranking of core EP-TF genes based on edge counts. **(F)** Thirty-five EP-TF genes in the PPI network calculated using the degree algorithm. **(G)** Dependency scores across the top genes with high edges in NB cells. BP: biological process; CC: cell component; EP-TF: epigenetic and transcriptional; GO: gene ontology; HR-NB: high-risk NB; MF: molecular function; NB: neuroblastoma; PPI: Protein-protein interaction.

We next investigated a total of 1,113 NB-dependent EP-TF genes, guided by three main criteria: 1) significantly upregulation in HR-NB tumor tissues (GSE12460, GSE13136 and GSE16476) as compared to normal adrenal gland tissues (GSE3526, GSE7307 and GSE8514) in the GEO datasets (Supplementary Figures S2A–D); 2) significantly upregulation in 24 NB cell lines (GSE28019) versus neural crest cells (GSE14340) in the GEO datasets (Supplementary Figure S2E); and 3) highest expression in NB from a pan-cancer viewpoint according to the cancer cell encyclopedia dataset (Supplementary Figure S2F). From this, we identified 35 genes of interest (Figure 3A). To further elucidate the role these 35 pivotal EP-TF genes in NB, we conducted a functional enrichment analysis, revealing that peptidyl-lysine modification, cohesion complex and nucleosome binding were the most significant terms in the GO-BP, GO-CC and GO-MF terms, respectively (Figures 3B–D). Furthermore, a PPI analysis revealed that the hub genes in NB included *SMARCA5, RUVBL1, MYCN,* and *SSRP1*, among others, as illustrated in Figures 3E,F. Additionally, NB cells demonstrated a significant reliance on these EP-TF genes, as evidenced by elevated negative dependency scores (Figure 3G).

### A ten-gene prognostic signature is developed based on 35 EP-TF genes in NB

**FIGURE 4:**
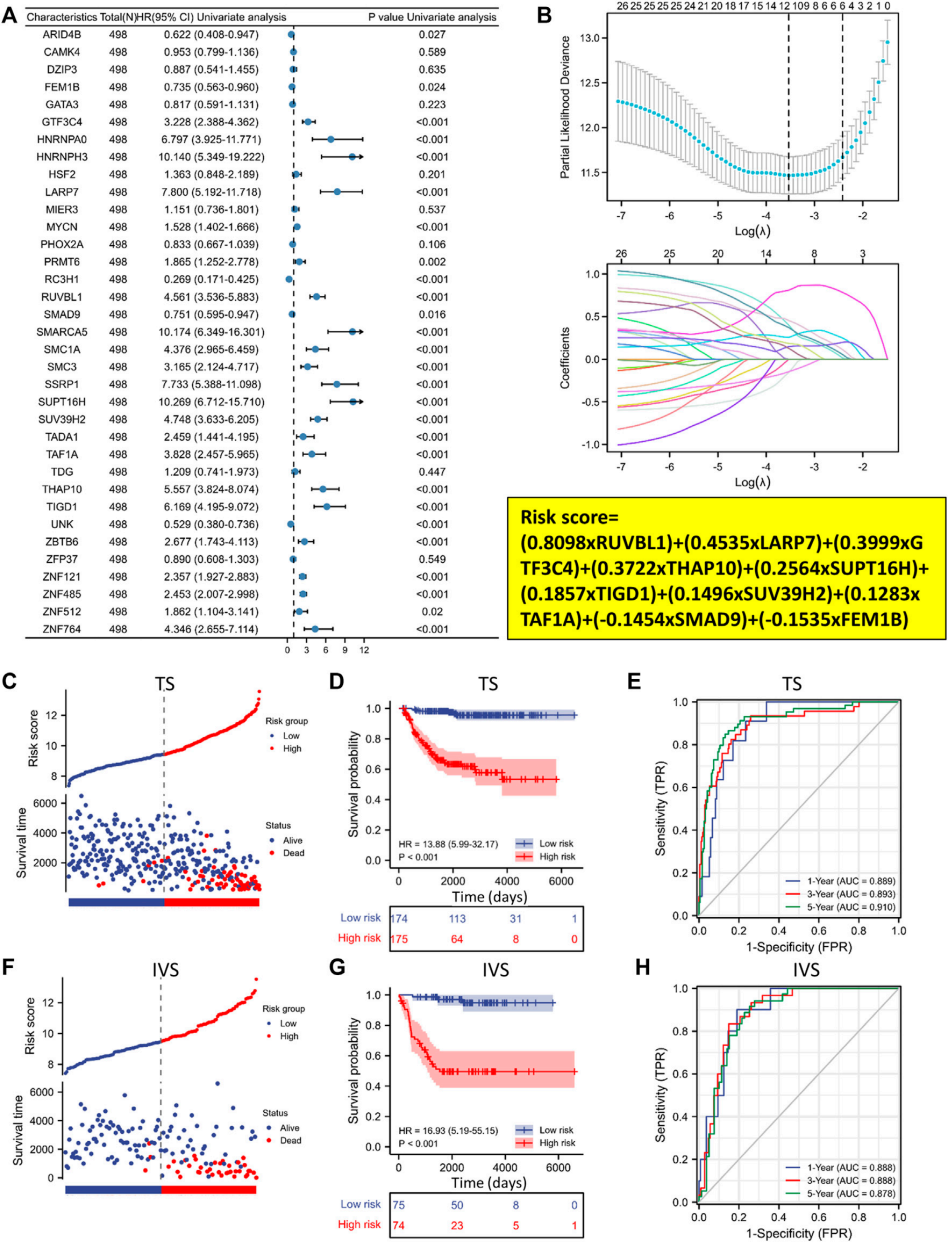
A ten-gene prognostic signature is developed based on the 35 EP-TF genes. **(A)** Univariate Cox regression analysis of the HRs and *p* values of 35 EP-TF genes. **(B)** A LASSO regression screen employed in the TS showing 10 of 27 candidate genes at the least deviance (upper panel) and coefficients of genes at different λ levels (lower panel). **(C–E)** In the TS, the risk score and survival distribution **(C)**, Kaplan-Meier curve **(D)** and time-dependent ROC curve **(E)** were evaluated, with risk groups determined by the ten-gene prognostic model. **(F–H)** In the IVS, the risk score and survival distribution **(F)**, Kaplan-Meier curve **(G)** and time-dependent ROC curve **(H)** were evaluated, with risk groups determined by the model. HR, hazard ratio; IVS, internal validation set; ROC: receiver operating characteristic; TS, training set.

We conducted a univariate Cox regression analysis with the GSE62564 dataset to evaluate the prognostic relevance of these 35 EP-TF genes. The analysis indicated significant correlations with OS for 27 genes (*p* < 0.05), identifying 22 as risk genes (e.g., *GTF3C4*, *HNRNPA0, HNRNPH3*) with HRs >1 and 5 as protective genes (e.g., *ARID4B, FEM1B, RC3H1*) with HRs <1 (Figure 4A). Applying the LASSO algorithm to the 27 genes in TS led to the selection of ten genes (*RUVBL1*, *LARP7*, *GTF3C4*, *THAP10*, *SUPT16H*, *TIGD1*, *SUV39H2*, *TAF1A*, *SMAD9* and *FEM1B*) for constructing a risk signature (Figure 4B, upper panel). The derived LRS was calculated from the LASSO coefficients (Figure 4B, lower panel, LRS= (0.8098x*RUVBL1*) + (0.4535x*LARP7*) + (0.3999x*GTF3C4*) + (0.3722x*THAP10*) + (0.2564x*SUPT16H*) + (0.1857x*TIGD1*) + (0.1496x*SUV39H2*) + (0.1283x*TAF1A*) + (−0.1454x*SMAD9*) + (−0.1535x*FEM1B*).

We further investigated the clinical significance of the 10-gene risk signature in NB patients. Using the median LRS, patients were divided into low- and high-risk categories within both the TS and IVS. Notably, the results demonstrated that a significant increase in mortality was linked to higher risk scores (Figures 4C, F). Additionally, survival rates were significantly lower than in the high-risk group for TS and IVS (Figures 4D, G). ROC curve analyses for 1, 3, and 5-year prognostic risk scores were performed, demonstrating the high predictive accuracy of the risk signature (area under curve >0.850 in all ROC curve analyses) in delineating OS in NB patients across TS and IVS (Figures 4E, H). In conclusion, the newly established prognostic model demonstrated robust performance in predicting outcomes in NB patients based on the GSE62564 dataset.

### External public datasets validate the EP-TF gene signature

**FIGURE 5:**
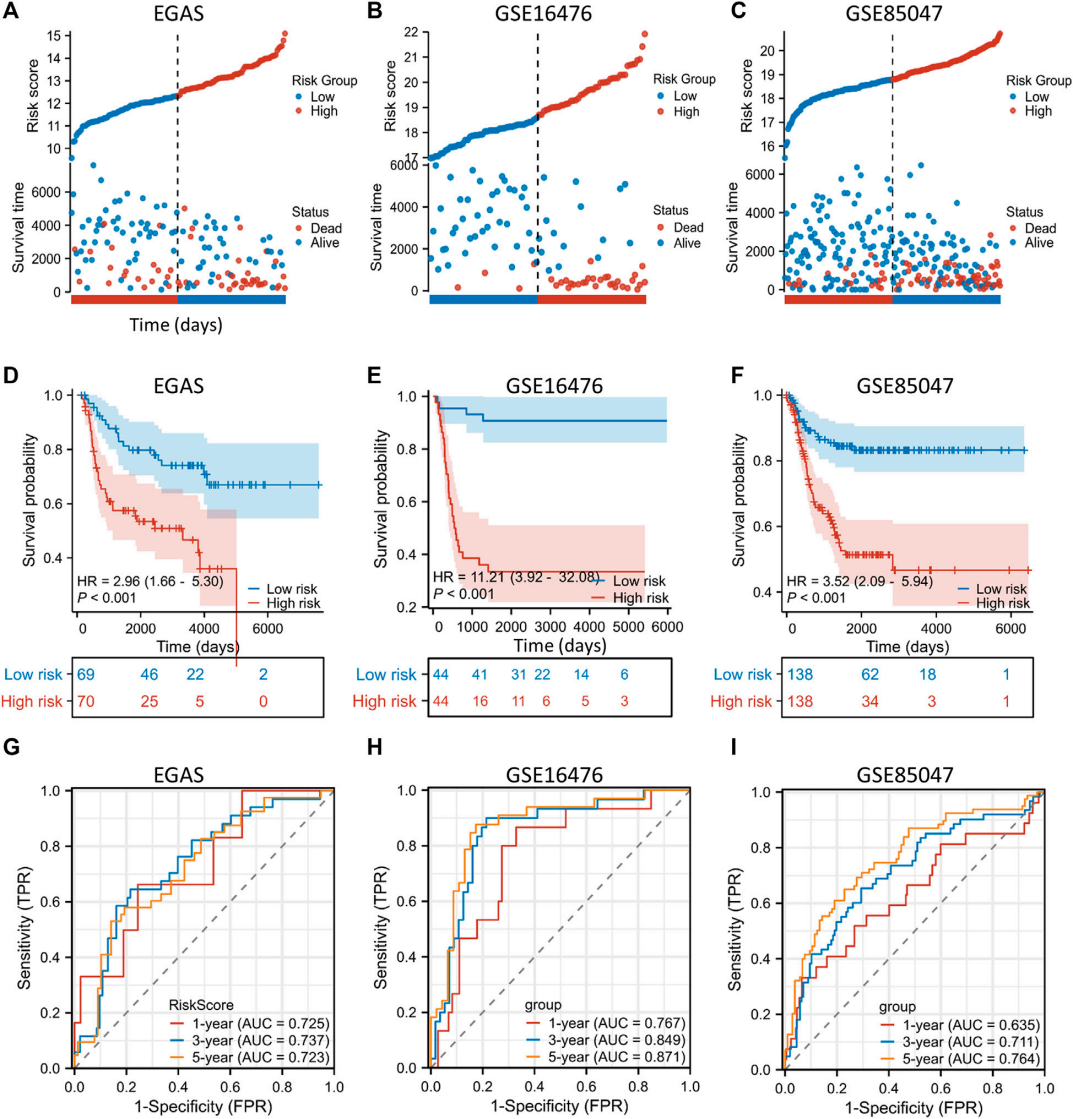
External validation of the gene signature is conducted using public datasets. **(A–C)** In the EGAS **(A)**, GSE16476 **(B)** and GSE85047 **(C)** datasets, risk scores and survival distributions were assessed using risk groups determined by the ten-gene prognostic model. **(D–F)** In the EGAS **(D)**, GSE16476 **(E)** and GSE85047 **(F)** datasets, Kaplan-Meier curves were generated using risk groups determined by the ten-gene prognostic model. **(G–I)** In datasets EGAS **(G)**, GSE16476 **(H)** and GSE85047 **(I)**, time-dependent ROC curves were assessed using risk groups determined by the ten-gene prognostic model. ROC: receiver operating characteristic.

To ascertain the generalizability of the EP-TF gene signature across diverse NB cohorts, we incorporated three external datasets for validation. These datasets encompassed one RNA-seq dataset, EGAS, and two microarray datasets (GSE16476 and GSE85047). The LASSO risk coefficients were utilized, and these three external datasets were subjected to a validation process. The performance of the 10-gene prognostic model was good, as evidenced by risk stratification plots (Figures 5A–C), Kaplan-Meier survival analyses (Figures 5D–F) and ROC curve evaluations (Figures 5G–I). Moreover, we examined the gene expression of 10 EP-TF genes in different INSS groups (Supplementary Figures S3A, C, E) and constructed a correlation matrix to elucidate potential gene interactions (Supplementary Figures S3B, D, F). In brief, the EP-TF gene model demonstrated robust applicability across diverse NB cohorts.

### NB tumors from SCH validate the EP-TF gene signature

**FIGURE 6:**
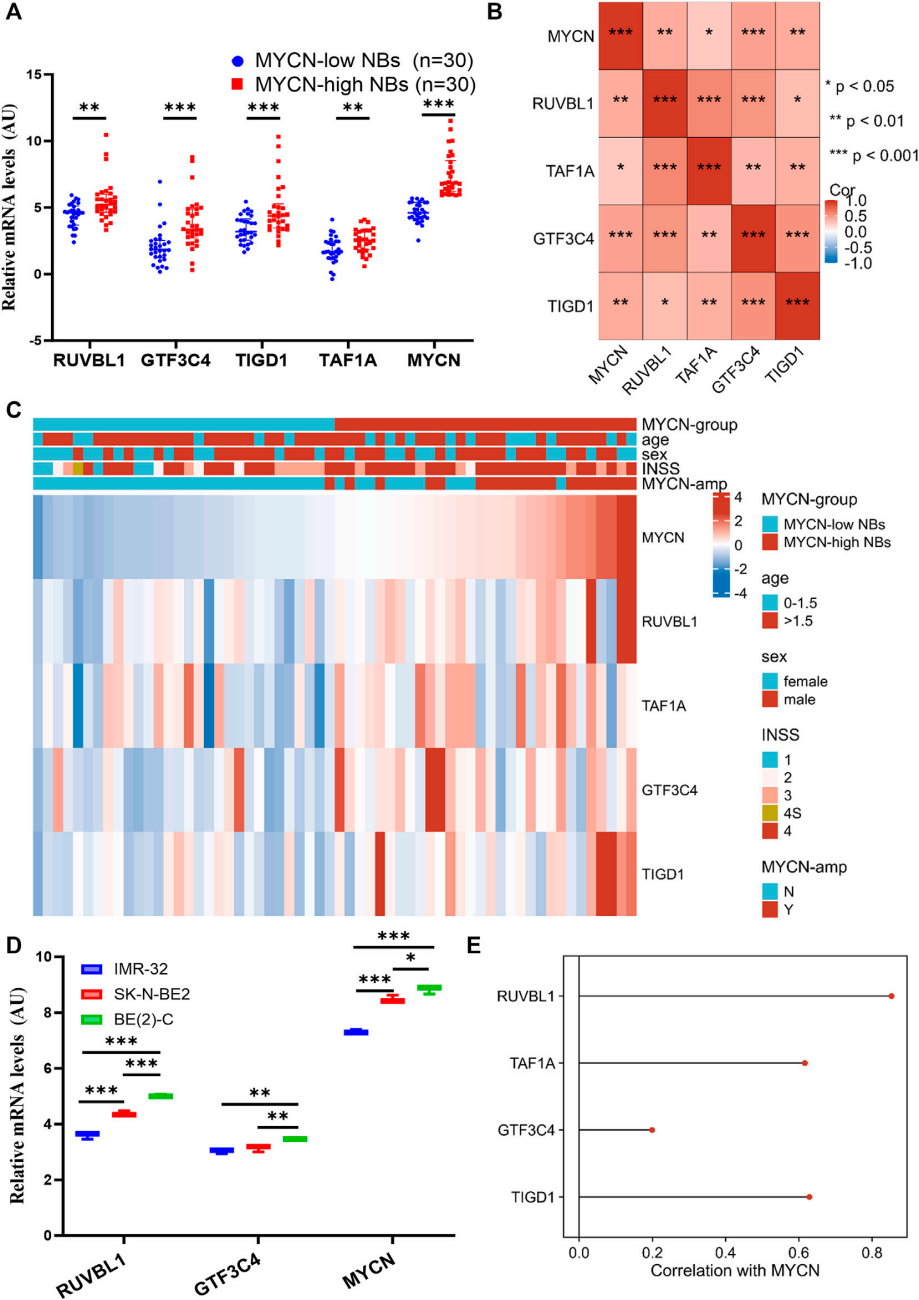
The EP-TF gene expression pattern of NB tumors in our center and NB models. **(A)** Transcriptional levels of representative EP-TF genes (*RUVBL1*, *GTF3C4*, *TIGD1* and *TAF1A*) and well-known NB-associated genes (*MYCN*) across NB tissue samples based on median *MYCN* levels from the SCH cohort (n = 60 in total), as quantified by Q-RT-PCR assays. **(B)** A matrix showing gene-gene correlation values among representative EP-TF genes in NB tumors from SCH. **(C)** The heatmap showing the profile of clinical characteristics and the EP-TF model in NB tumors from SCH. **(D)** Transcriptional levels of representative EP-TF genes (*RUVBL1* and *GTF3C4*) and well-known NB-associated genes (*MYCN*) across *MYCN*-amplified NB cells, as quantified by Q-RT-PCR assays. **(E)** Lollipops chart showing correlations between *MYCN* and EP-TF genes (*RUVBL1*, *GTF3C4, TAF1A* and *TIGD1*) in four primary NB PDX models. HR-NB: high-risk NB; INSS: international neuroblastoma staging system; PDX: patient-derived xenograft; Q-RT-PCR: quantitative reverse transcription polymerase chain reaction; *MYCN*-amp: *MYCN*-amplified; SCH: Shanghai Children’s Hospital. *p* < 0.05 is shown as *, *p* < 0.01 as ** and *p* < 0.001 as ***.

After validation with external datasets, we evaluated the precision of the EP-TF signature in NB tissue samples from our center, SCH. We examined the transcriptional levels of key tumor-dependent genes (*RUVBL1*, *GTF3C4*, *TIGD1,* and *TAF1A*) between groups categorized by *MYCN* expression levels (low vs. high, n = 30 each) using Q-RT-PCR. The results showed augmented levels of EP-TF genes and *MYCN* in the *MYCN*-high group (Figure 6A). Additionally, a positive gene-gene correlation matrix from the SCH cohort highlighted similarities with diverse NB cohorts (Figure 6B; Supplementary Figures S3B, D, F). Additionally, we depicted the gene expression profiles and clinical characteristics within the SCH cohort (n = 60 in total), thus illustrating the robust performance of the EP-TF gene signature (Figure 6C).

By exploring the traits of the EP-TF signature in NB cells, our Q-RT-PCR analyses revealed that *RUVBL1* and *GTF3C4* transcriptional levels were elevated in NB cells with high *MYCN* expression (Figure 6D). Parallel findings were observed upon examining public datasets (Supplementary Figures S4A, B), revealing a positive correlation between *MYCN* and most EP-TF risk genes (Supplementary Figures S4C, D). Moreover, we found positive correlations between *MYCN* and EP-TF genes in primary NB PDXs (Figure 6E). Collectively, these findings derived from our NB tissue specimens and cell lines confirm the validity of the EP-TF gene signature and suggest a potential interaction between MYCN and EP-TF genes.

### MYCN serves a potential upstream regulator of EP-TF genes

**FIGURE 7:**
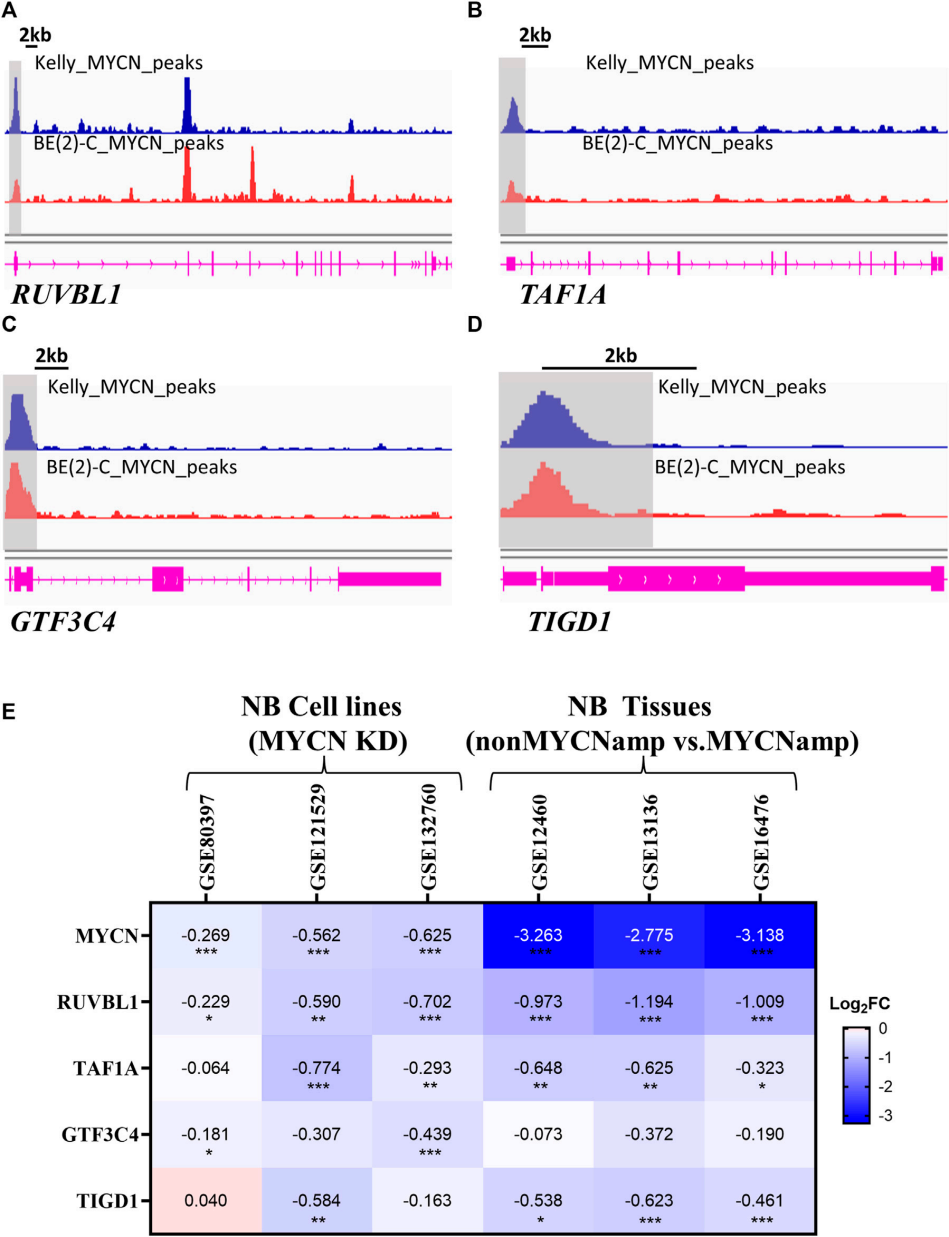
MYCN serves as an upstream regulator of EP-TF genes. **(A–D)** ChIP-seq data showing the binding region of MYCN to *RUVBL1* **(A)** *, TAF1A* **(B)** *, GTF3C4* **(C)** and *TIGD1* **(D)** in the promoter. **(E)** The Heatmap showing changes in the expression of EP-TF genes in NB cells after MYCN knockdown and NB tumors in non-MYCN-amp tissues. ChIP-seq: chromatin immunoprecipitation sequencing; FC: fold change; non*MYCN*-amp: non*MYCN*-amplified. *p* < 0.05 is shown as *, *p* < 0.01 as ** and *p* < 0.001 as ***.

Prior analyses have demonstrated a robust correlation between *MYCN* and EP-TF genes, suggesting that MYCN could function as an upstream driver in the regulation of these genes. To investigate this regulatory mechanism, we analyzed anti-MYCN ChIP-seq data, which revealed that MYCN bound to the promoter regions of *RUVBL1, TAF1A, GTF3C4* and *TIGD1* in MYCN-amplified BE (2)-C and Kelly cells (Figures 7A–D). Then, we examined *MYCN* knockdown datasets to assess the impact of MYCN binding on promoter regions of EP-TF genes and found a general downregulation of EP-TF genes following MYCN knockdown. (Figure 7E). In addition, the EP-TF genes exhibited significantly reduced levels in nonMYCN-amplified NB tumors compared to MYCN-amplified counterparts (Figure 7E). Collectively, these data suggest that MYCN might bind to the promoter regions of EP-TF genes and thus enhance their expression in NB.

### The EP-TF model can be utilized in the biological and clinical evaluation of NB

**FIGURE 8:**
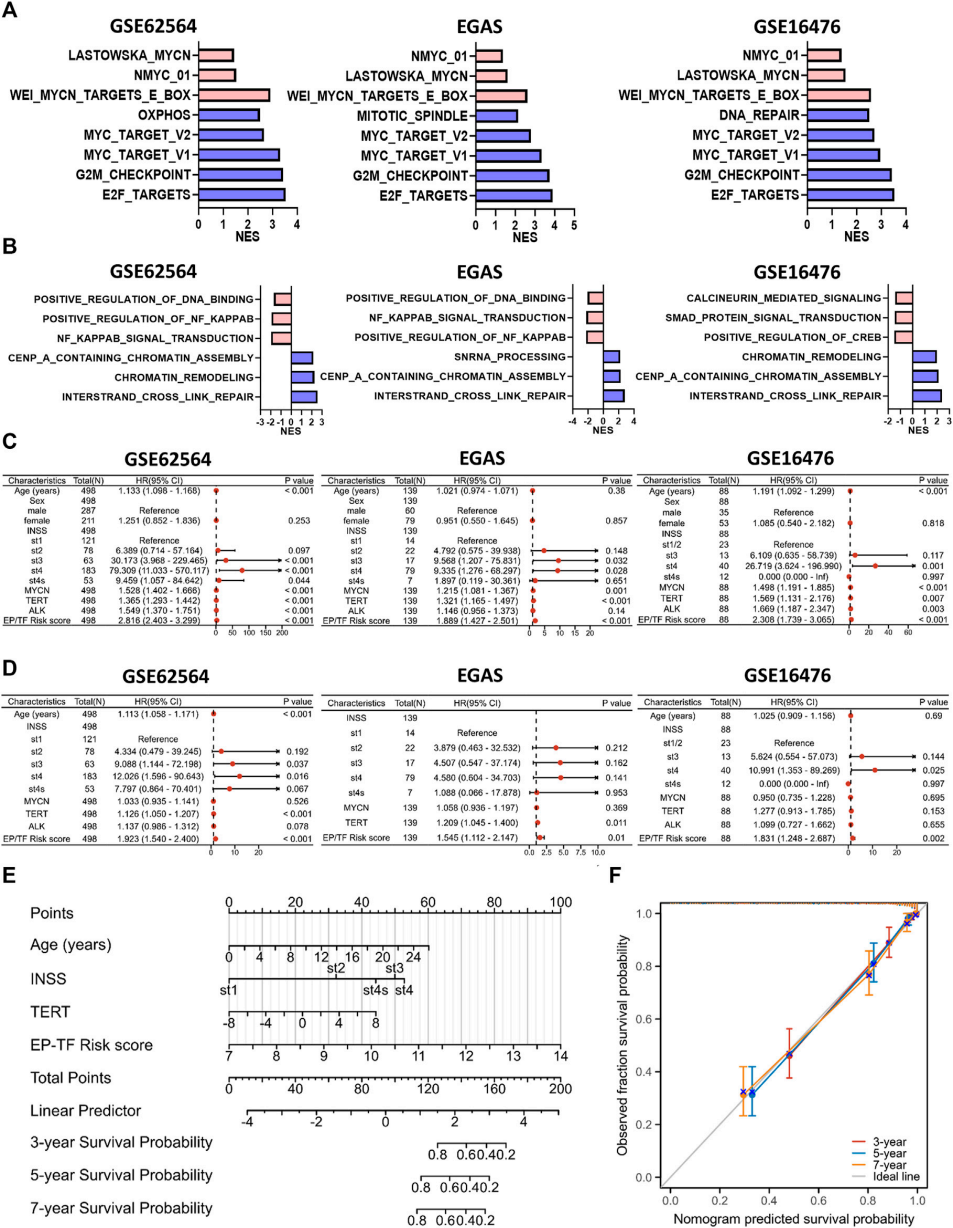
The EP-TF model facilitates straightforward application in the evaluation of the biological and clinical status of NB. **(A)** The top five hallmark gene sets and *MYCN*-related gene sets enriched in the high-risk group obtained by GSEA in GSE62564, EGAS and GSE16476. **(B)** Three representative EP-TF gene sets enriched in the high-risk group (blue columns) and low-risk group (red columns) obtained by GSEA in GSE62564, EGAS and GSE16476. **(C, D)** Univariate **(C)** and multivariate **(D)** Cox regression for sex, age, INSS stage, *ALK*, *MYCN*, and *TERT* levels and the risk score according to the ten-gene model in GSE62564, EGAS and GSE16476. **(E)** Nomogram for clinical practitioners in GSE62564. **(F)** Calibration plot (with 100 subjects per group and 800 resampling iterations) for the nomogram in GSE62564.

We employed the EP-TF model to stratify patients with NB into high-risk and low-risk groups based on the median LRS from the GSE62564, EGAS and GSE16476 datasets. Comprehensive GSEA was performed across the three NB datasets using the “Hallmark”, “*MYCN*-related” and “EP-TF” gene sets (Figures 8A,B). The HR-NBs were positively enriched for oncogenic (blue columns in Figure 8A) and *MYCN*-related (red columns in Figure 8A) phenotypes. Moreover, the HR-NBs were positively enriched for the EP-TF terms of “DNA interstrand crosslink repair” and “chromatin remodeling” (blue columns in Figure 8B), and negatively enriched for “NF-κB signal transduction” (red columns in Figure 8B).

Additionally, we explored the clinical characteristics of NB patients, including age, sex, INSS stage and *MYCN, ALK* and *TERT* levels, within the context of our model. Through univariate and multivariate Cox regression analyses of the GSE62564, EGAS and GSE16476 datasets, we evaluated whether the EP-TF signature independently predicted patient outcomes. Univariate analysis indicated that sex (*p* > 0.05 in all NB cohorts), age (*p* > 0.05 in the EGAS cohort) and *ALK* status (*p* > 0.05 in the EGAS) were not risk factors, unlike INSS stages, *MYCN* and *TERT* levels and the risk score of the 10-gene model (HR > 1, *p* < 0.05 in all NB cohorts; Figure 8C). Multivariate analysis confirmed that the EP-TF model risk score was a strong independent prognostic factor across all NB cohorts (HR = 1.923, *p* < 0.001 in GSE62564; HR = 1.545, *p* = 0.01 in EGAS; HR = 1.831, *p* = 0.002 in GSE16476) (Figure 8D). Furthermore, we developed a nomogram for clinical prediction that integrates age, INSS stage, *TERT* level and the EP-TF risk score to offer clinicians a quantitative tool for predicting 3-, 5-, and 7-year OS probabilities for patients with NB. The nomogram (Figure 8E) assigned points to each prognostic parameter for individual patients, where a higher total point score indicated an inferior prognosis, accompanied by a well-executed calibration plot (Figure 8F).

### MEKis might serve as synergistic agents in combination with immunotherapy in HR-NBs characterized by EP-TF aberrations

**FIGURE 9:**
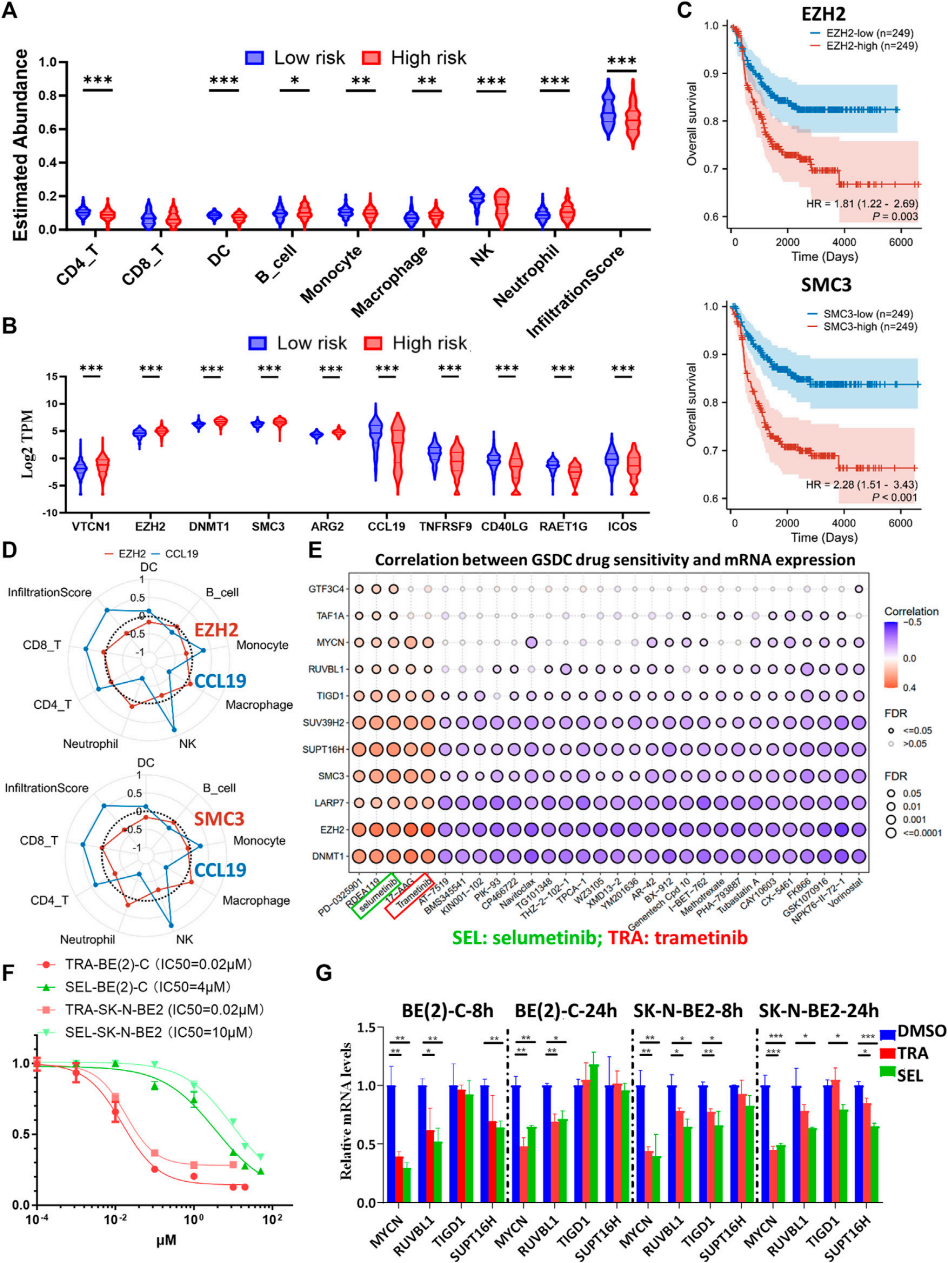
Comprehensive analysis of the immune landscape in NB patients and investigation of potential therapeutic targets. **(A, B)** Violin plots depicting the variances in leukocyte infiltration **(A)** and immune-related marker expression **(B)** among different risk groups, as determined by the ten-gene model in GSE62564. **(C)** Kaplan-Meier curves comparing the high and low expression groups of *EZH2* (upper panel) and *SMC3* (lower panel) in GSE62564. **(D)** Radar maps showing the correlations between immune cell abundance and *EZH2* (upper panel), *SMC3* (lower panel) and CCL19 (used as an immune-rich control). **(E)** A matrix demonstrating the relationship between GDSC drug sensitivity and mRNA expression of key genes (including *MYCN*, *EZH2*, *SMC3*, *DNMT1*, and seven representative genes from the prognostic model), highlighting the potential efficacy of MEK inhibitors. **(F)** Dose-response curves of TRA and SEL in BE (2)-C and SK-N-BE2 NB cells. **(G)** Transcriptional alterations in *MYCN*, *RUVBL1*, *TIGD1* and *SUPT16H* after eight or 24 h of TRA or SEL treatment, as quantified by Q-RT-PCR assays. GDSC: Genomics of Drug Sensitivity in Cancer; HR, hazard ratio; Q-RT-PCR: quantitative reverse transcription polymerase chain reaction; SEL: selumetinib; TRA: trametinib. *p* < 0.05 is shown as *, *p* < 0.01 as ** and *p* < 0.001 as ***.

Immunotherapy has emerged as a highly promising treatment modality for NB (Verhoeven et al., 2022), thus, we utilized the ImmuCellAI online tool for single-sample GSEA to explore immune cell infiltration in patients with NB. Our results revealed a diminished presence of immunoactive CD4^+^T cells, dendritic cells (DCs), B cells, monocytes, and NK cells and an overall reduced total infiltration score in the HR-NB subtype defined by the EP-TF gene signature (Figure 9A). We also analyzed the gene profiles of anti-inflammatory and pro-inflammatory cytokines (http://biocc.hrbmu.edu.cn/TIP/). This enabled us to visualize an increase in representative immunosuppressive markers and a decrease in representative immunoactive markers (Figure 9B). Previous immunotherapy studies advocated the blockade of immunosuppressive targets as an effective strategy (e.g., inhibition of *PD-L1* (Okla et al., 2020) or *CTLA4* (Ascierto et al., 2011)); thus, our focus was directed toward immunosuppressive markers highly expressed on NB cells (*EZH2*, *SMC3* and *DNMT1*). One of our previous studies indicated the role of *DNMT1*. In this regard, we analyzed the role of *EZH2* and *SMC3* and showed that escalated expression of *EZH2* or *SMC3* correlated with poorer OS (Figure 9C). Furthermore, we examined the correlations between *EZH2* or *SMC3* (using CCL19 as a positive control) and various immune cells. Our results suggested that *EZH2* might exert an immunosuppressive effect on DCs, monocytes and NK cells, and that *SMC3* might have immunosuppressive impact on DCs, NK cells and CD4^+^T cells (Figure 9D).

In addition, we conducted a drug sensitivity analysis through the GDSC database using the GSCALite online tool. The analysis included key biomarkers including *MYCN* and immunosuppressive indicators (*EZH2*, *SMC3* and *DNMT1*), along with other representative risk genes from the EP-TF model. The results highlighted the potential efficacy of MEKis, particularly trametinib (TRA) and selumetinib (SEL), as evidenced by their positive sensitivity correlation with *MYCN*, *EZH2*, *SMC3*, *DNMT1* and other risk genes (Figure 9E). Next, we evaluated the effects of TRA and SEL on *MYCN*-amplified BE (2)-C and SK-N-BE2 cell lines, observing that the IC50 value for TRA was 0.02 μM for both cell lines, whereas for SEL, it was 4 μM for BE (2)-C and 10 μM for SK-N-BE2 (Figure 9F). Next, we treated NB cells with 1 μM TRA or 10 μM SEL for 8 or 24 h, which resulted in a notable reduction in the mRNA levels of *MYCN* and key EP-TF risk genes (*RUVBL1*, *TIGD1* and *SUPT16H*) in both BE (2)-C and SK-N-BE2 cells (Figure 9G). The results of external datasets also indicated that TRA inhibited *MYCN, RUVBL,* and *TAF1A* expression (Supplementary Figure S5A). Interestingly, we carefully checked the PDX data and found that retinoic acid and JQ1 exerted similar inhibitory effects on MYCN and EP-TF genes (Supplementary Figure S5B). Taken together, these results suggest that decreased numbers of DCs and NK cells are associated with elevated levels of *EZH2* and *SMC3* in the HR-NB group. Moreover, MEKi has emerged as a promising therapeutic agent for targeting oncogenic and EP-TF-dysregulated pathways.

## Discussion

HR-NB poses a great challenge in pediatric oncology. This study conducted a comprehensive analysis using CRISPR-Cas9 knockout screening combined with transcriptomics, revealing 35 EP-TF genes that exhibit high expression levels in NB tissue and are critical for tumor viability. Following univariate analysis and *MYCN* exclusion, 27 out of 35 genes were selected through LASSO screening. The developed model incorporated 10 EP-TF genes, including *RUVBL1*, *LARP7*, and *GTF3C4* among others, demonstrating substantial prognostic value across diverse NB cohorts. The EP-TF signature underscores the complex biological underpinnings of NB, highlighting the relevance of genes involved in cell cycle regulation, *MYCN*-associated signaling pathways and chromatin remodeling—all of which are associated with adverse outcomes and immunosuppression. MEKis have emerged as promising candidates for targeting oncogenic pathways disrupted by EP-TF dysregulation. The development and validation of the EP-TF prognostic model optimize existing molecular classification schemes for NB, thereby paving the way for novel drug target identification.

Previous studies have revealed epigenetic and transcriptional dysregulation in NB, with altered EP-TF mechanisms including DNA methylation, histone modification, noncoding RNA regulation, super-enhancer modification, bromodomain regulation and chromatin remodeling, particularly in *MYCN*-amplified NB cells (Jimenez et al., 2023). Furthermore, *MYCN* is a critical transcription factor that accelerates cell cycle progression by targeting *CDK4, CHK1, ID2, MCM, MYBL2, SKP2*, etc. and influences a broad transcriptional network that remains under investigation (Matthay et al., 2016). Consequently, we conducted a comprehensive analysis and constructed an EP-TF model expressed as LRS= (0.8098xRUVBL1) + (0.4535xLARP7) + (0.3999xGTF3C4) + (0.3722xTHAP10) + (0.2564xSUPT16H) + (0.1857xTIGD1) + (0.1496xSUV39H2) + (0.1283xTAF1A) + (−0.1454xSMAD9) + (−0.1535xFEM1B).

*RUVBL1* has both DNA-dependent ATPase and DNA helicase activities and engages in several multisubunit transcriptional and protein complexes, contributing to ATP-dependent remodeling and histone modification. Liu et al. demonstrated that the recruitment of *RUVBL1*/Tip60 complexes activates NRF1, thereby promoting colorectal cancer progression (Liu et al., 2022). However, the role of *RUVBL1* in NB requires further investigation. *LARP7* is linked to the 7SK small nuclear ribonucleoprotein complex, which suppresses a cyclin-dependent kinase crucial for initiating transcription elongation via RNA polymerase II (Eichhorn et al., 2018). The role of *LARP7* in cancer remains controversial, as it functions either an activator (Tong et al., 2022) or suppressor (Ji et al., 2014) in cancer, but its impact on NB remains undocumented. *GTF3C4*, a part of transcription factor in the TFIIIC complex and that is present in the mitochondria and nucleoplasm, is presumed to facilitate enzyme activation and DNA binding (Taranu et al., 2015), although research on its role in cancer is sparse. *THAP10*, characterized by an N-terminal Thanatos-associated domain with a zinc finger motif similar to DNA-binding domains, shows potential repression in breast cancer (De Souza Santos et al., 2008) and leukemia (Li et al., 2017), but no studies have addressed its function in NB.

The Facilitates Chromatin Transcription Complex, which functions as a histone chaperone with the ability to remodel chromatin, is composed of the *SSRP1* and *SUPT16H* subunits (Mo et al., 2023). Carter et al. reported that the complex and *MYCN* form a forward feedback loop in NB cells, which is essential for their sustained high expression. Inhibition of the complex by the curaxin CBL0137 markedly impedes tumor growth *in vivo* (Carter et al., 2015). *TIGD1* belongs to the tigger subfamily within the human pogo superfamily of DNA-mediated transposons, but its specific function remains unclear. Several bioinformatic analyses have suggested its involvement in cancer progression (Wu et al., 2023; Zhang et al., 2023). *SUV39H2* is involved in histone methylation (H3-K9 specific) and chromatin assembly (Padeken et al., 2022) and is linked to colorectal cancer proliferation and metastasis (Shuai et al., 2018), but its function in NB has yet to be clarified (Tong et al., 2013). *TAF1A*, part of the RNA polymerase I complex, is involved in assembling the RNA polymerase I preinitiation complex and has been linked to cell proliferation in cervical cancer (Wang et al., 2021), but its role in NB remains unexplored.

Interestingly, our previous and current investigations revealed that low-stage or non-HR-NB tumors exhibit increased expression of *SMAD9*, despite extensive experimentation to validate the *SMAD9*-*MYCN* positive feedback loop in *MYCN*-amplified NB cells (Tan et al., 2022). This finding suggests a variable function of *SMAD9* in *MYCN*-amplified versus non-*MYCN*-amplified NB cells, meriting additional research to uncover the specific mechanisms involved. Manford et al. reported that the *CUL2-FEM1B* complex ubiquitylates *FNIP1*, thereby augmenting mitochondrial reactive oxygen species, supporting our finding that *FEM1B* might exert a protective effect on NB tumorigenesis by elevated reactive oxygen species in immune cells (Manford et al., 2020).

We observed reduced populations of CD4^+^ T cells, monocytes, DCs and NKs in HR-NBs based on the EP-TF model, consistent with previous findings of diminished immune cells including T cells, mono-DC traffic cells and NK cells in *MYCN*-amplified NBs (Yang et al., 2023). A recent study suggested the critical role of CXCR3^+^ monocytes in immunotherapy (Kaczanowska et al., 2024) and we also detected a significant decrease in CXCR3 in HR-NBs (data not shown). In addition, Jimenez et al. highlighted the epigenetic modulatory therapeutic potential in enhancing immunogenicity (Jimenez et al., 2023). The efficacy of MEKi against most EP-TF risk genes in NB further aligned with previous studies, suggesting that MEK inhibition could mitigate EP-TF related oncogenic alterations in high-grade ovarian cancer (Machino et al., 2023) and lung cancer (Li et al., 2019) and counteract resistance to chemoimmunotherapy. Earlier reports have indicated that MEK inhibition promotes degradation of *MYCN* (Yaari et al., 2005), with recent studies focusing on EP-TF mechanisms of resistance to MEKis (Coggins et al., 2019; Pilgrim et al., 2023).

Interestingly, except for MEKis, we identified the potential inhibitory role of retinoic acid and JQ1 in *MYCN* and EP-TF genes. Zimmerman et al., 2021. reported that retinoic acid can reprogram the enhancer landscape, which results in downregulation of MYCN expression. Shi et al. (2022) concluded that JQ1 serves as a BRD4 inhibitor and downregulate MYCN expression. These results are consistent with our analysis, indicating the upstream regulatory role of MYCN.

Some limitations should be noted. Our study was largely based on retrospective data, which did not compensate for the need for prospective validation. Moreover, we did not employ single-cell or single-nucleus transcriptomic approaches to validate immune cell subtype distinctions across NB subtypes. Our analysis primarily focused on NB tissue datasets with limited validation, and thus the specific causative mechanisms among EP-TF genes in NB cells necessitate further elucidation through additional biological experimentation. Further investigations are required in the future.

In conclusion, the theoretical significance of our study stems from its enhancement of the molecular understanding of NB, particularly in how EP-TF anomalies contribute to disease progression and patient prognosis. From a clinical perspective, our model introduces a novel tool for risk stratification and personalized treatment, offering the potential for more targeted and efficacious therapeutic strategies for NB patients. Future research priorities include the prospective validation of the EP-TF model, the investigation of immunotherapy tailored to the tumor microenvironment and the performance of clinical trials to ascertain the effectiveness of specific drug targets, especially MEKis. By addressing these issues, further research can augment the understanding and management of NB.

## Conclusion

Our comprehensive CRISPR-Cas9 knockout screening in conjunction with transcriptomic analysis reveals an epigenetic and transcriptional gene signature that optimizes risk stratification in neuroblastoma patients and paves the way for identifying novel drug targets.

## Data Availability

The original contributions presented in the study are included in the article/Supplementary Material (Supplementary Tables S1–S4), further inquiries can be directed to the corresponding authors. The datasets presented in this study can be found in online repositories. The names of the repositories and accession numbers can be found in the article/Supplementary Material (Supplementary Table S5).

## Abbreviations

BP, Biological process; CC, Cell component; ChIP-seq, Chromatin immunoprecipitation sequencing; DC, Dendritic cell; EGAS, EGAS00001001308; EP-TF, Epigenetic and transcriptional; EVS, External validation set; FBS, Fetal bovine serum; GDSC, Genomics of Drug Sensitivity in Cancer; GSEA, Gene set enrichment analysis; HR, Hazard ratio; HR-NB, High-risk NB; IVS, Internal validation set; LASSO, Least absolute shrinkage and selection operator; LRS, LASSO risk score; MEKi, MEK inhibitor; MF, Molecular function; NB, Neuroblastoma; NK, Natural killer cell; OS, Overall survival; P/S, Penicillin/Streptomycin; PPI, Protein-protein interaction; RNA-seq, RNA sequencing; ROC, Receiver operating characteristic; SCH, Shanghai Children’s Hospital; SEL, Selumetinib; TRA, Trametinib; TS, Training set.
