# Supplementary material for: CRISPR-Cas9 screening develops an epigenetic and transcriptional gene signature for risk stratification and target prediction in neuroblastoma
Source: Front Cell Dev Biol. 2024 Aug 8;12:1433008. doi: 10.3389/fcell.2024.1433008 (PMC11338898; doi:10.3389/fcell.2024.1433008)
Supplement: Supplementary file 2 [file DataSheet1.docx]

**Supplemental Figures**


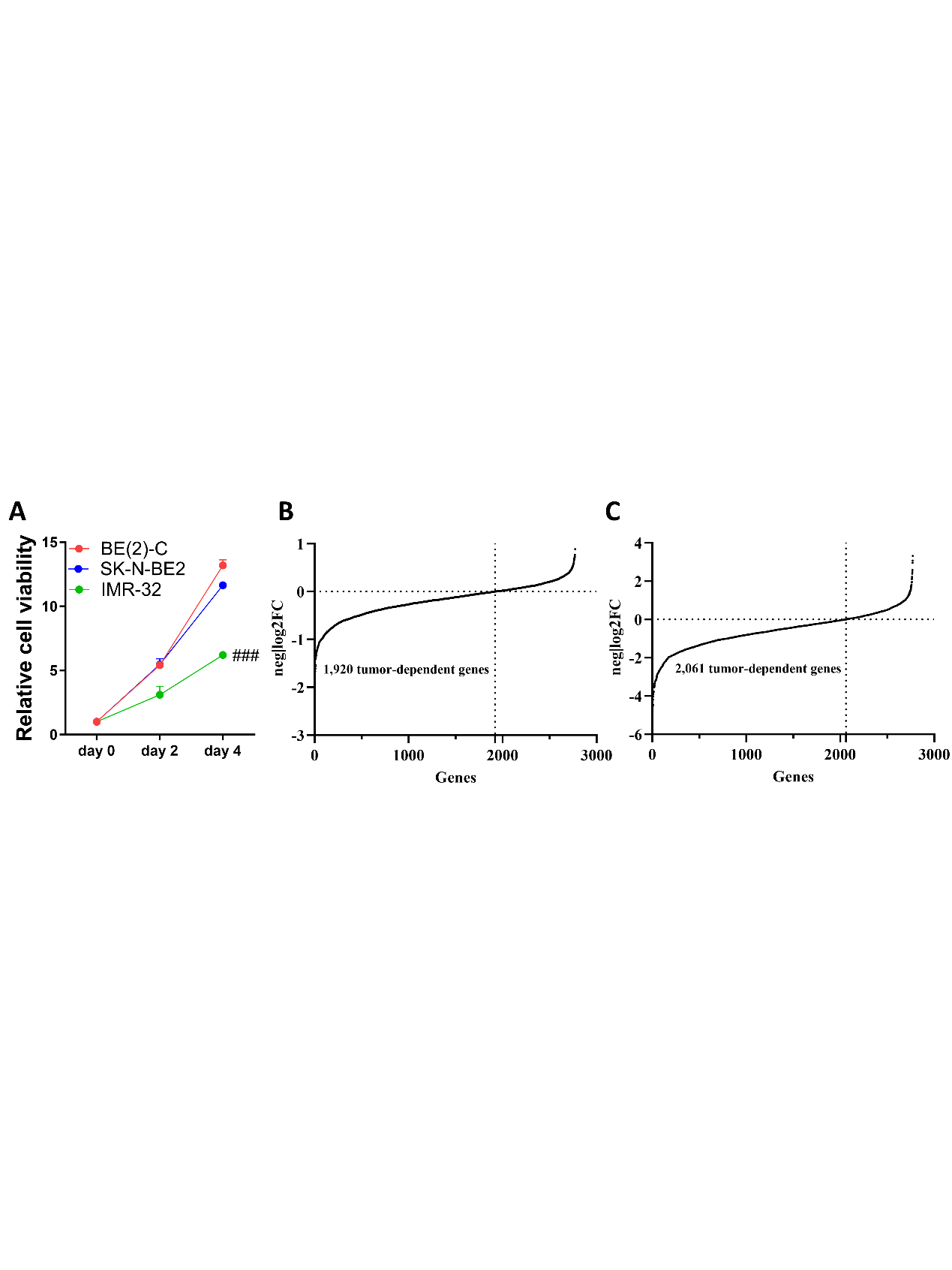


**Figure S1. Identification of NB-dependent EP-TF regulatory genes via CRISPR-Cas9 knockout screening.**

(**A**) Growth curve of MYCN-amplified NB cell in vitro. (**B-C**) Results from CRISPR-Cas9 screening of Cas9-transfected BE(2)-C cells, conducted both in vitro (B) and in vivo (C). ^#^ indicated the comparison between BE(2)-C and IMR32. P<0.05 was shown as ^#^, P<0.01 as ^##^ and P<0.001 as ^###^. NB: neuroblastoma.


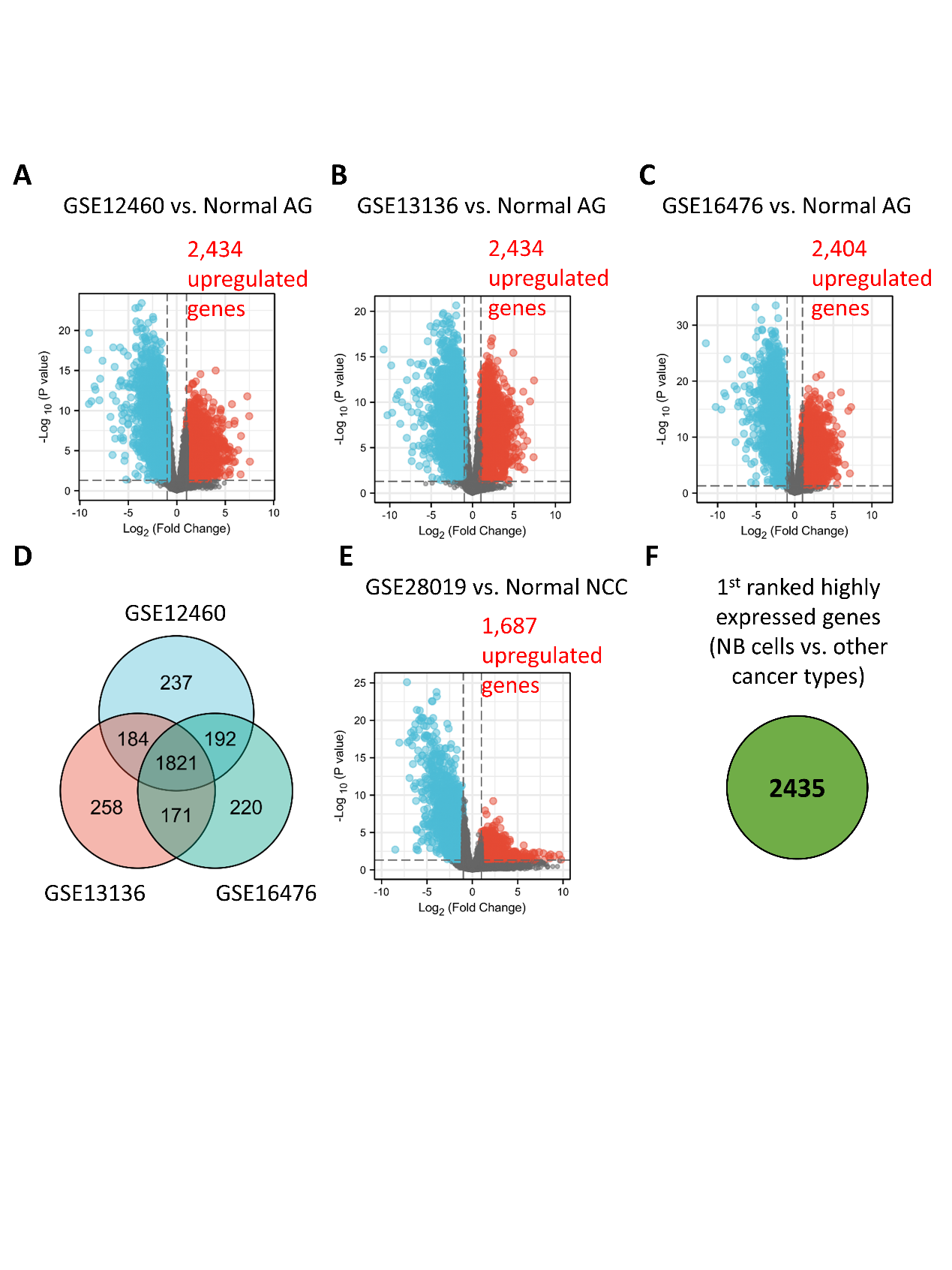


**Figure S2. Transcript analysis identifies DEGs in diverse NB tissues and cells.**

(**A-C**) Volcano plots showing DEGs between high-risk NB tissues and normal AGs across three datasets: GSE12460 (A), GSE13136 (B) and GSE16476 (C). (**D**) Veen diagram displaying genes common to all three datasets. (**E**) Volcano plot for DEGs between high-risk NB cells and NCCs. (**F**) A pan-cancer analysis from DepMap dataset revealing the top highly expressed genes in NB. AGs: adrenal glands; DEGs: differential expressed genes; NB: neuroblastoma; NCCs: neural crest cells.


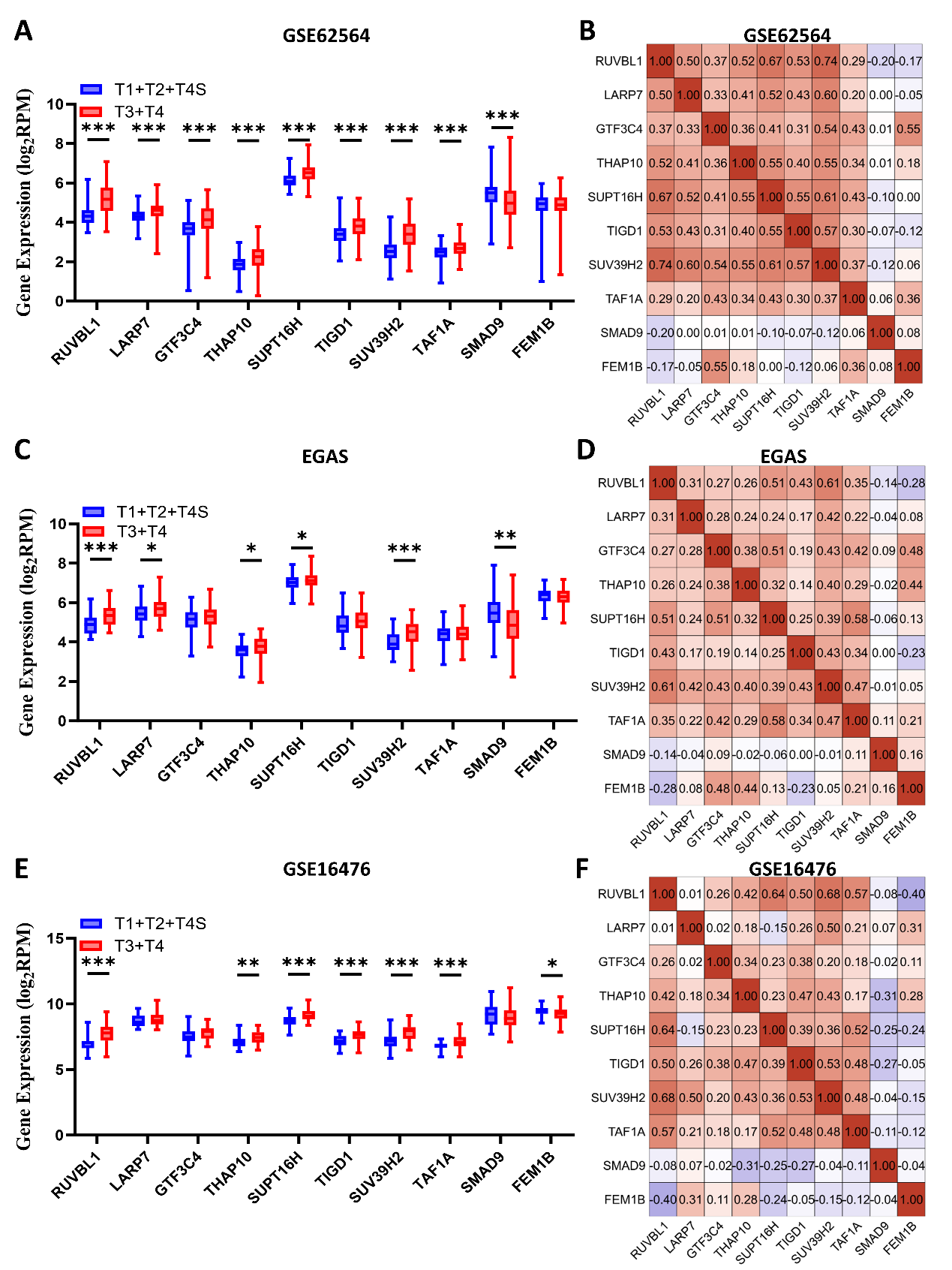


**Figure S3. Gene expression profiles and correlation matrices in NB tissues indicate the predictive value of the EP-TF gene signature.**

(**A, C, E**) Box plots depicting gene expression differences between higher (T3+T4) and lower (T1+T2+T4S) stages NB tissues according to INSS, across three datasets: GSE62564 (A), EGAS (C) and GSE16476 (E). (**B, D, F**) Correlation matrices showing gene-gene potential interactions for the datasets of GSE62564 (B), EGAS (D) and GSE16476 (F). INSS: internation neuroblastoma staging system; NB: neuroblastoma.


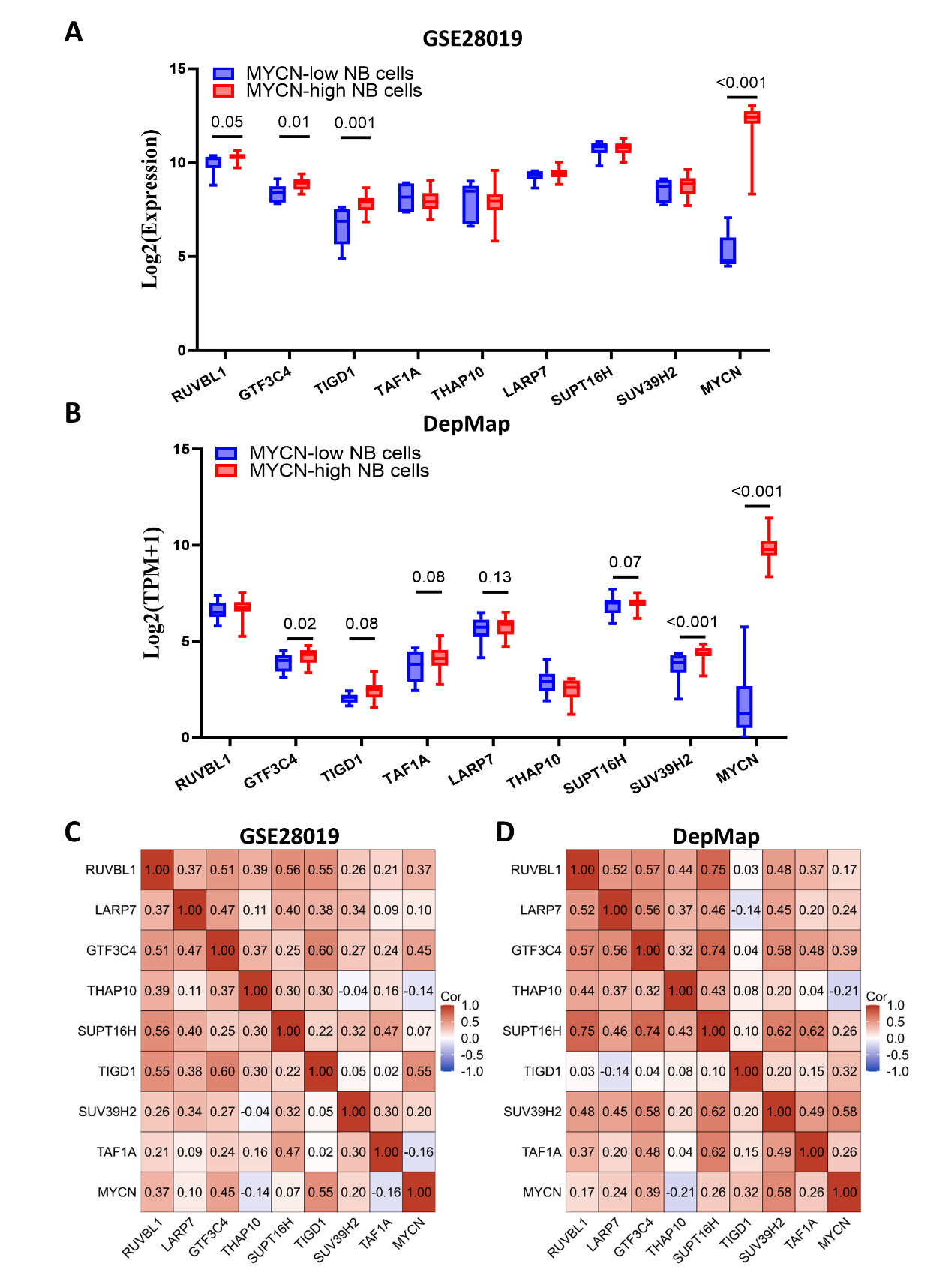


**Figure S4. Gene expression profiles and correlation matrices in NB cells highlight predictive value of the EP-TF gene signature.**

(**A, B**) DEG profiles between MYCN-high and MYCN-low NB cells (based on the median expression of MYCN) as shown in GSE28019 (A) and the DepMap (B) datasets. (**C, D**) Correlation matrices illustrating gene-gene correlations within the datasets of GSE28019 (C) and DepMap (D). DEGs: differential expressed genes; NB: neuroblastoma.


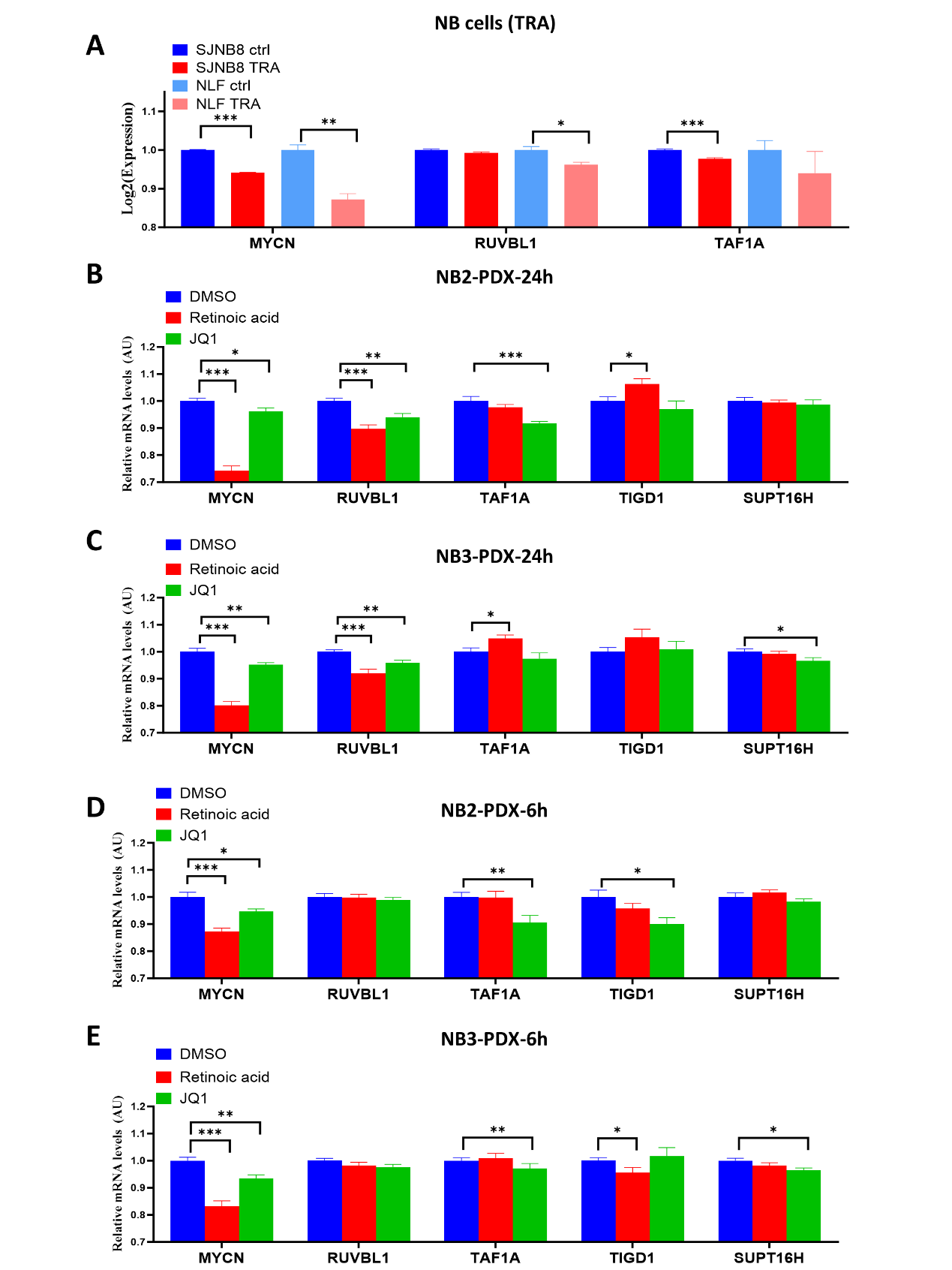


**Figure S5. Further investigation of potential therapeutic targets.**

(**A**) Transcriptional alterations in *MYCN*, *RUVBL1* and *TAF1A* after 6 hours of TRA treatment in SJNB8 cells and 72 hours of TRA treatment in NLF cells. (**B-C**) Changes in *MYCN*, *RUVBL1*, *TAF1A, TIGD1 and SUPT16H* transcription after 24 hours of retinoic acid and JQ1 treatment in NB2-PDX cells (B) and NB3-PDX cells (C). (**D-E**) Changes in *MYCN*, *RUVBL1*, *TAF1A, TIGD1 and SUPT16H* transcription after 6 hours of retinoic acid and JQ1 treatment in NB2-PDX cells (D) and NB3-PDX cells (E). TRA: trametinib; PDX: patient derived xenograft; NB: neuroblastoma. P<0.05 is shown as *, P<0.01 as ** and P<0.001 as ***.
